# Migratory birds benefit from urban environments in a highly anthropized Neotropical region

**DOI:** 10.1371/journal.pone.0311290

**Published:** 2025-01-24

**Authors:** Rodrigo Pacheco-Muñoz, Adrián Ceja-Madrigal, Jorge E. Schondube

**Affiliations:** 1 Instituto de Investigaciones en Ecosistemas y Sustentabilidad, Universidad Nacional Autónoma de México, Morelia, Michoacán, México; 2 Posgrado en Ciencias Biológicas, Universidad Nacional Autónoma de México, Coyoacán, Ciudad de México, México; UFERSA: Universidade Federal Rural do Semi-Arido, BRAZIL

## Abstract

Land use change from wildlands to urban and productive environments can dramatically transform ecosystem structure and processes. Despite their structural and functional differences from wildlands, human-modified environments offer unique habitat elements for wildlife. In this study, we examined how migratory birds use urban, productive, and wildland environments of a highly anthropized region of Western Mexico known as “El Bajío”. We used Generalized Linear Models to compare species richness, abundance, and the functional traits of migratory bird assemblages among these three environments. Results revealed differences in species richness, composition, and the functional traits of migratory birds among environments. Regardless of wildlands showing medium to high levels of human disturbance, they presented the highest species richness and abundance of migratory birds, with urban environments presenting the lowest values. Insectivorous and granivorous birds were dominant in the migratory bird assemblages of the three environments. The migratory bird assemblages of productive environments had more grassland granivorous birds. In contrast, insectivorous birds with dense habitat preferences and short culmen lengths dominated the urban bird assemblage. Migratory bird assemblages in productive and urban environments showed similar species richness and abundance of insectivorous birds, but they differ in their composition. Our results reveal that urban trees allowed cities to function as simplified forests, showing that the urban environment has the untapped potential to support complex assemblages of migratory birds. To promote migratory birds in human-modified landscapes, we must maintain complex vegetation areas that allow birds with diverse functional traits to overwinter in urban and productive environments.

## Introduction

Historically, land use change has been primarily driven by expanding human-productive systems over areas of native vegetation (i.e., wildlands from here on), with one-third of Earth’s land ice-free surfaces having been transformed [1, 2]. However, over the past century, urbanization has become the land use change driver with the fastest increase rate, typically transforming productive areas located close to human settlements [3]. Nevertheless, wildlands can also be affected by urbanization. These modifications have resulted in urban regions (*sensu* Forman [4]), constituted by a complex landscape mosaic that includes productive and wildland environments that interact with the flows and processes generated by the human settlements. Hence, urban environments transform ecosystem processes over large spatial scales [5, 6], becoming crucial for understanding the social, economic, and political drivers influencing land use change [7].

Land use change by urbanization is characterized by replacing non-human elements of the environment with built-in impermeable cover and infrastructure, creating a different ecological system [8]. The urban ecosystem presents a high human population, and the buildings, infrastructure, and resources required to maintain it [9]. In addition to human-made structures, vegetation is a crucial element of urban ecosystems [10]. Urban vegetation tends to be composed of a limited number of fragmented remnants of native vegetation, enriched with de novo green areas where humans plant a diverse array that includes both native and exotic species [11, 12]. The cultural tendency to maintain open grass lawns with low density and diversity of shrubs generates a simplified lower stratum in the urban vegetation [11]. Additionally, trees tend to grow faster and larger inside cities [13].

Other environmental characteristics particular to urban ecosystems include altered water regimes, with a year-round relatively secure availability of water, regardless of local seasonal changes in precipitation [14]; a surplus of food resources [15]; higher temperatures given the urban heat island effect [16]; and the presence of constant human activity that is related to noise, light, and other forms of pollution [17, 18]. As a result, replacing productive and wild environments with urban areas offers a novel array of ecological opportunities for the regional pool of species, including those present only on a seasonal basis [19, 20]. However, their capacity to capitalize the opportunities offered by the urban environment vary according to each species’ life history and functional traits [20]. While we are beginning to understand how resident birds interact with cities, we lack information on urbanization’s effects on migratory avifauna [14].

The Nearctic-Neotropical migratory landbirds (hereafter called migratory birds) are a polyphyletic group of species exclusive to the American continent [21]. Their annual cycle consists of a breeding stage in the Nearctic region during spring and summer and a non-breeding overwintering stage in the Neotropical region from late autumn until early spring [22–24]. Between those stages, they conduct southbound and northbound long-distance movements [23, 24]. The survival of migratory birds is notably dependent on the quality of their overwintering habitats, given that they spend most of their annual cycle in them [22, 24]. Most populations of migratory bird species have been declining, with the transformation of wildlands to productive and urban environments in their overwintering region representing the main threat to the survival of their populations [24, 25].

Overwintering migratory birds use a broad range of habitats, and can occur in greater abundance in disturbed habitats than in undisturbed [26–28]. Yet, the benefits of habitat disturbance apparently occur at intermediate levels and are damped at higher intensities [26]. The transformation of wildlands to productive environments can dramatically reduce the richness of migratory bird species, generating an average loss of species of 20% [29]. Productive environments promote migratory bird species with greater tolerance to open habitats that are less dependent on the vegetation structure, present lower foraging heights and are granivorous [27, 30, 31]. While patterns of migratory birds species richness and abundance have been studied in the past in wildlands and productive environments of the Neotropics (see works and reviews [22, 24, 27, 32–35]), they have been poorly studied in urban environments (but see [36–43]).

Our limited knowledge of urban migratory bird assemblages indicates that they present lower species richness relative to wildlands [36]. Urban migratory birds are mainly foliage-gleaning insectivore species that are closely associated with trees [36, 38, 42, 44]. Given these birds’ narrow association with trees, Neotropical urban environments may function as regional refuges for them [14, 42, 43]. The relevance of urban environments as refuges for migratory birds could be especially important when its surrounding areas present a high level of human modification, harsh environmental conditions, or limited critical resources [43].

In this study, we assessed the role played by urban areas to maintain the regional pool of migratory birds in a highly anthropized region of Western Mexico known as El Bajío. To do this, we contrasted migratory birds’ taxonomic and functional diversity among the three main environmental conditions present in this region: urban, productive (agriculture), and wildlands (native vegetation; following MacGregor-Fors & Vázquez [45]). We classified the regional landscape using this approach because it allows us to separate environmental conditions by combining habitat structure and human activities [45]. We expect that wildlands will act as the main reservoir for the regional pool of these birds [29, 46]. At the same time, urban environments will present lower species richness and abundance of migratory birds than the two non-urban environments [29, 36]. Additionally, we expect that migratory bird assemblages will show contrasting functional traits (morphological and behavioral) among environments, with a higher functional diversity in non-urban environments [29, 47]. We expect productive environments to promote species with a granivorous diet or open habitat preferences [27, 30, 31], while the urban environment will allow the presence of insectivorous species associated with forested habitats [38, 42].

## Methods

### Study area and sampling locations

**Fig 1:**
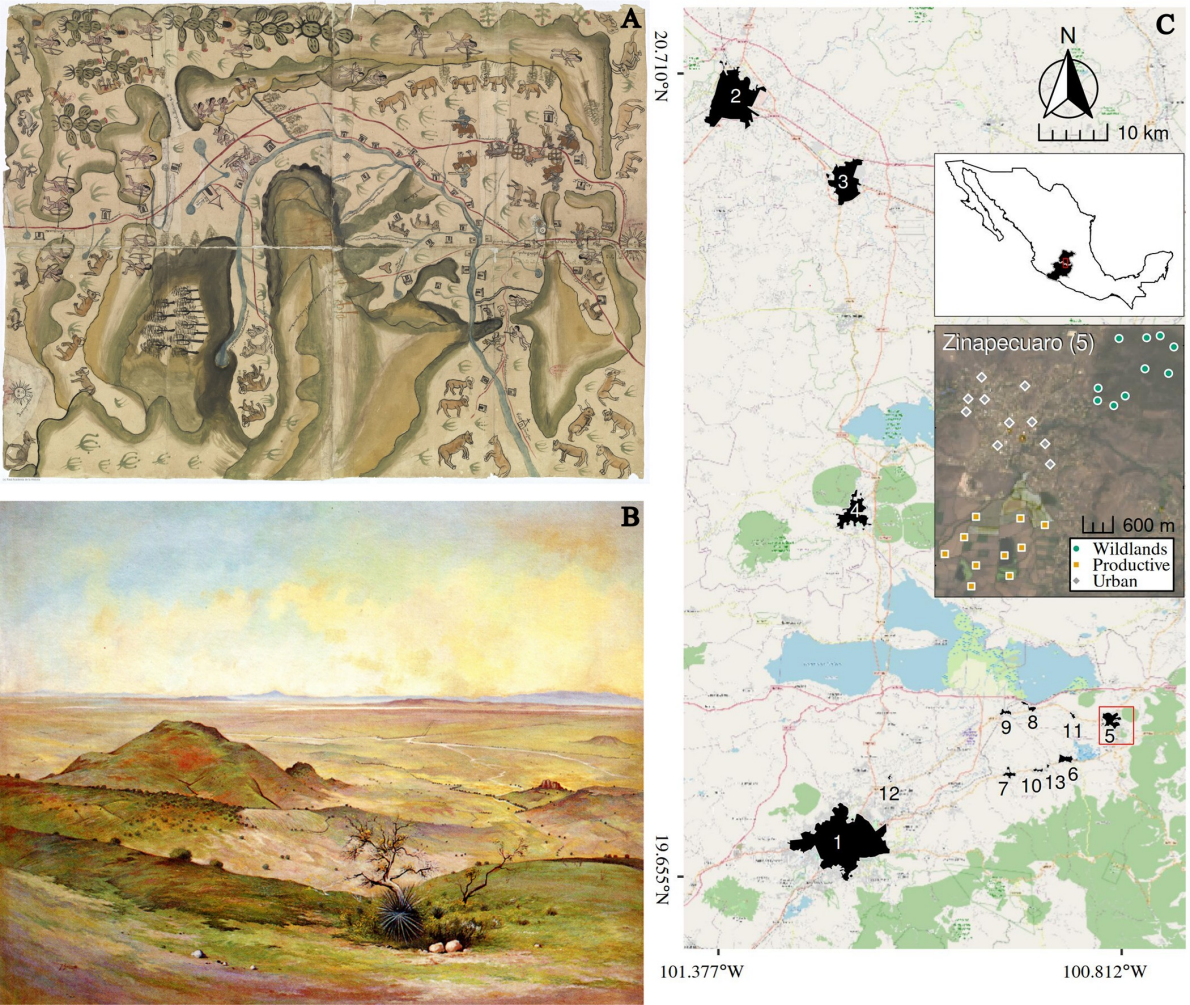
El Bajío is a biocultural region that presents a continuous human occupation with agricultural activities for the last 3,000 years. As a result it presents a complex landscape mosaic characterized by high levels of human disturbance. A) The cartographic codex “Mapa de las villas de San Miguel y San Felipe de los Chichimecas y el pueblo de San Francisco Chamacuero” (1578–1580) shows that most of the territory (~70%) was already occupied by crops and cattle production by the end of the XVI century. B) Painting “Paisaje del Bajío” (1960) by the landscape artist Jesús Gallardo™ Carrillo (Republished from [53] under a CC BY license, with permission and courtesy from the owners of “Jesús Gallardo”™, original copyright 1960). This painting depicts the moment in the mid 20th at which most wildland remnants disappeared from the lowlands of the region, intensifying the long-term anthropogenic transformation that El Bajío has undergone. This generated a landscape with large open areas of lowlands used for agricultural purposes and a few remnant areas of native vegetation present on the hills. C) Map of our studied localities in El Bajío (Data by OpenStreetMap under ODbL were used to create the map [54]). We sampled thirteen human settlements: Morelia (1), Irapuato (2), Salamanca (3), Moroleon-Uriangato (4), Zinapécuaro (5), Queréndaro (6), Indaparapeo (7), Francisco Villa (8), Belisario Domínguez (9), San Lucas Pío (10), José María Morelos (11), San Pedro de los Sauces (12) and Colonia Guadalupe (13). The inserted Landsat-8 satellite image shows the location of sampling points for Zinapecuaro to exemplify their general arrangement of sampling sites in the three environments for each location. In addition to each surveyed human settlement (gray), we surveyed the wildlands (green) and productive (orange) environments that surrounded them.

We conducted our study in the region known as "*El Bajío*". El Bajío is an urban region (*sensu* Forman [1]) which, apart from presenting shared eco-physiographic characteristics [48], delimits a Mexican territory with a common socioeconomic history [49]. It is located within the biocultural region of Western Mexico (*sensu* Schöndube [37]) in the Mexican states of Guanajuato and Michoacán [50]; Longitudes: 101.4077–100.8015° W; Latitudes: 19.63878–20.73814° N; Fig 1). It presents an altitude range from 1721 to 2181 masl [51], with a dry-winter subtropical highland climate (Koppen climate type: Cwb; average annual precipitation: 723.7 ± 92.5 mm; mean annual temperature: 17.8 ± 1.1°C; maximum temperature: 32.4°C; minimum temperature: 2.8°C [52]).

El Bajío has been an important agricultural region for the last 3,000 years. This process started after the introduction of maize to the region by the Chupicuaro culture ([37]; Fig 1A). Later in the 17th century, it was intensely deforested under Spanish colonial rule to use the wood in the mine´s furnaces, and to open space for cattle grazing areas and cereal production ([39]; Fig 1A). In the mid 20th century, government subsidies and programs incentivized agro-industrial farming that are still present ([36, 39]; Fig 1B). Finally, the North American Free Trade Agreement (NAFTA) promoted the industrialization and urbanization of the region after 1994 [55]. This urban growth has occurred mainly on agricultural areas. Wildlands at our studied region are scattered patches of subtropical xerophytic scrubland, deciduous oak, pine-oak forests, and transition stages among these vegetation types [56]. This environment covers 19.6% of the territory of our study area [57]. Wildlands patches have the most complex vegetation structure in the region, mainly occurring in steep slopes and ravines, where agricultural activities are limited by topography and water availability [55, 57]. The most common native species of trees and shrubs include: *Bursera fagaroides*, *Casimiroa edulis*, *Celtis pallida*, *Ehretia latifolia*, *Fraxinus uhdei*, *Ipomea murucoides*, *Opuntia tomentosa*, *Pinus devoniana*, *P*. *leiophylla*, *P*. *pseudostrobus*, *P*. *laevigata*, *Quercus castanea*, *Q*. *rugosa*, *Salix humboldtiana*, *Stenocereus queretaroensis*, *Vachellia farnesiana*, *Vackellia pannatula*, and *Yucca filifera* [56]. Human pressure on this environmental condition includes illegal logging; wood gathering for fuel; free-ranging livestock (goats and cows); human-generated fires; the introduction of exotic plant species (*Casuarina equisetifolia*, *Eucalyptus* spp., *Morus alba*, and *Schinus molle*); and land use change into agricultural and urban conditions [58]. Most wildlands remnants do not have a protection status, with only a few small sized protected areas existing in the region (i.e., Cerro del Punguato, Cerro de Arandas, Siete Luminarias; CONABIO [59]).

The productive environment comprises agricultural activities (crops, greenhouses, plantations, and livestock [55]), covering 73.3% of the territory [57]. Most cultivars are produced using irrigation (open channels with flooding or aspersion systems), with seasonal crops being produced on slopes during the rainy season [56]. Traditional crops include corn, sorghum, wheat, legumes, berries, alfalfa, and vegetables like broccoli, cauliflower, asparagus, and tomatoes [55, 60]. However, in the last decade, several of these cultivars have been replaced with blue agave (*Agave tequilana*) plantations to produce tequila and mezcal. Crop fields tend to be divided by living fences of both native and exotic tree species (*Casuarina equisetifolia*, *Celtis pallida*, *Eucalyptus* spp., *Fraxinus uhdei*, *Morus alba*, *Salix humboldtiana*, *Schinus molle*, *Vachellia* spp.; personal observation R. P-M; [61]).

Finally, the urban environment is constituted by human settlements of different sizes (~0.28 km^2^—~115 km^2^) and human densities (~1,067 hab/km^2^—~9,687 hab/km^2^; [62]) that cover 6.8% of our study area [57]. The human settlements included in our study were: Colonia Guadalupe, San Pedro de los Sauces, José María Morelos, San Lucas Pío, Belisario Domínguez, Francisco Villa, Indaparapeo, Queréndaro, Zinapécuaro, Moroleon-Uriangato, Salamanca, Irapuato, and Morelia (Fig 1). The majority of them can be classified as residential towns with agricultural pursuits. However, the large urban centers (size ≥ 10 km^2^; Salamanca, Moroleon-Uriangato, Irapuato, and Morelia) include important commercial and industrial activities [55]. Residential areas display mostly one or two-story houses with little gardens or patios, while several-story buildings are rare and only present in large urban centers. Streets tend to be well illuminated, with exposed electric and communication infrastructure (poles and cables). This often limits the presence of trees and their size when they are planted on the streets [63]. Urban vegetation is scattered inside urban settlements and includes both native (i.e. *Celtis pallida*, *Cupressus lusitanica*, *Fraxinus uhdei*, *Salix humboldtiana*, and *Taxodium mucronatum*) and exotic species (*Casuarina equisetifolia*, *Eriobotrya japonica*, *Eucalyptus* spp., *Ficus benjamina*, *Grevillea robusta*, *Jacaranda mimosifolia*, *Ligustrum lucidum*, *Magnolia grandiflora*, *Schinus molle*). It is important to acknowledge that urban vegetated areas present an oversimplified understory relative to wildlands [43].

### Bird surveys

We surveyed migratory birds in thirteen localities of El Bajío. Our sampling focused on the southern area of this region. We chose this geographic area because it presents the larger cover of wildlands at El Bajío. Each sampling locality included an urban environment and its adjacent productive, and wildland environments (see our survey example in Fig 1). To determine bird species richness and abundances, we conducted 10 min long point-counts with distance estimations [64]. One observer performed all the bird surveys (A. C.-M.). We recorded birds during a period of 4 hours (from sunrise to mid morning; ~7:00–11:00), targeting the peak activity period of birds. While performing our surveys, we recorded only the birds using the habitat (not those that only flew over the vegetation). All migratory birds detected during the point-counts were included in our species richness analyses. Only migratory birds detected within a 40 m radius of the observer were included in our relative abundance analyses. The radius distance was selected based on the area that included ≥75% of all detections throughout our study (37.5 m radius). We sampled during the winter months after migratory birds had established their overwintering territories in this urban region (November 2018 to February 2019).

To conduct bird surveys, we delimited the areas occupied by each environment on the landscape at each location using a geographical information system [65]. We randomly deployed survey stations inside the delimited area of each environment per location, depending on its size (7–10 survey stations separated by a minimum distance of 250 m). In urban settlements larger than 10 km^2^ we sampled more survey stations (20 in Salamanca, Moroleon-Uriangato, and Irapuato, and 30 in Morelia). In those occasions, survey stations were located at a minimum distance of 500 m. As a result, the total number of sampling stations varied among environments (106 in wildlands, 130 in productive, and 175 in the urban environment). During field work we sampled one locality at a time, sampling a maximum of 10 sampling stations/day. Each sampling station was visited only once. Sampling was conducted on open public spaces or from public roads and streets. When we sampled private spaces we verbally asked for permission to conduct our work. We did not sample when these conditions were not met.

### Birds’ functional traits

Birds sampled were classified as migratory following Berlanga [66]. Additionally, bird species that presented both migratory and resident populations (i.e., *Troglodytes aedon*, *Polioptila caerulea*) were considered migratory if they were absent during summer or their abundance doubled during the winter in West Mexico (R. P-M. and J. E. S. unpublished data). We excluded migratory waterfowl and raptors from our analyses. Functional traits were classified into categorical or continuous traits. Categorical traits included: 1) their primary trophic guild (carnivore, frugivore, granivore, insectivore, nectarivore, omnivore, and scavenger; [67]), 2) their primary foraging behavior (hawking, excavating, gleaning, and hovering; [67]), and 3) their preferred habitat density (open, semi-open and dense habitats; [68]). Continuous traits included: culmen (mm), beak width (mm), beak depth (mm), tarsus length (mm), tail length (mm), hand-wing index, and body mass (g). We also included the Primary Foraging Stratum mainly used as a continuous variable in our analyses (1: ground, 2: understory, 3: mid-high, 4: canopy, and 5: aerial; [69]). These variables were collected from the AVONET database [68], except the Primary Foraging Stratum. We determined the value of this variable for each species based on the percentages of use for each stratum category in the Elton traits database [55]. We determine the value for each species as the strata at which it accumulated more than 50% of its time, going from the lowest to the highest stratum.

### Data processing and analysis

We contrasted the migratory bird assemblages among the three sampled environments of El Bajío region. We compared the assemblages’ species richness, abundance, structure, composition, and functional traits. We calculated migrant bird species richness by interpolating point-count incidence-based rarefactions to 106 points. This number represents the minimum point-count effort for one of our sampled environments (wildlands; [70]). We conducted this analysis using iNext package in R [71]. Estimated species richness values were calculated with asymmetric confidence intervals of 84%. These confidence intervals allowed us to determine non-overlapping comparisons with a *p*(ɑ) ≤ 0.05 [72]. We contrasted migrant bird abundance among habitat types using a negative-binomial generalized linear model. This family model was chosen over a Poisson one due to the overdispersion found in the latter. We performed pairwise Tukey tests for each model through the "emmeans" R package [73].

We also conducted analyses by trophic guild. We focused on the two dominant trophic guilds of migratory birds at our study region: insectivores and granivores (see below in the results section). Our trophic guild analyses included 1) a comparison of bird species richness, relative abundance and functional diversity for each trophic guild (insectivore or granivore) among environments; and 2) a comparison of abundances among the different groups that constitute the selected categorical traits (tropic guilds, primary foraging behavior and preferred habitat density) in the three environments. These comparisons were conducted similarly to those mentioned above for all migratory birds.

We evaluated the structure and composition of migratory bird assemblages by calculating the effort-based relative abundance of each species in each environment. We standardized all species’ relative abundances to individuals per point-count to achieve this (individuals/point-count). We used these abundance values to generate rank-abundance plots following Whittaker [74]. Briefly, a rank-abundance plot displays the assemblage evenness by ordering the species on the horizontal axis by their relative abundances from highest to lowest and displaying their relative abundances on the vertical axis. We used rank-abundance plots to compare the assemblage’s evenness among environments. We compared them using a linear model with the log10 transformed relative abundances as the response variable and the environment and their species ranks as interacting factors.

We assessed the migrant bird assemblage composition by calculating a Bray-Curtis dissimilarity index considering all locations [75]. We subsequently processed them by using a non-metric multidimensional scaling (MDS) on "vegan" R package [76]. Then we compared the assemblages among environments with an adonis test [76]. We considered the environment as the only factor, and we performed a subsequent pairwise posthoc test [76]. We also assessed each bird species association with the three environments by calculating phi coefficients with all recorded species [77]. The phi coefficient ranges from -1 to 1, where 0 represents no species association with the environment type. A positive coefficient reflects a positive association, and a negative coefficient reflects a negative one [77]. The obtained phi coefficient is independent of sample size and was calculated using "indicspecies" package in R [78].

We assessed differences in functional diversity among environments using multitrait Gower distance and community-weighted mean values (CWM) considering all functional traits (see above). These variables were weighted by the species’ relative abundances in each environment [79]. Given that the Gower distance values can give disproportional weight to certain variables, we calculated them and their "gawdis" R function following de Bello et al. [80]. Afterward, we used the calculated Gower distance to obtain the FEve, FDiv, and FDis functional diversity indexes [79, 81]. We calculated CWM with “FD” R package [82]. The functional diversity index values obtained from functional traits comprised of continuous values were compared using generalized linear models considering the environment as the only factor. Categorical functional traits were also contrasted with generalized linear models considering the environment and the categorical trait as two interacting factors. In both cases, we assessed the differences given by the model using a posthoc pairwise Tukey test by their estimated marginal means [73]. We assumed relevant contrasts at a *p*(ɑ) = 0.05.

## Results

We found a total of 43 migratory bird species in our studied Neotropical region. This species assemblage includes members of 13 families of birds located in the orders Passeriformes (12 families, 39 species) and Apodiformes (one family, four species). The most important migratory bird trophic guilds were insectivores (26 species) and granivores (9 species), followed by nectarivores and omnivores (4 species each).

**Fig 2:**
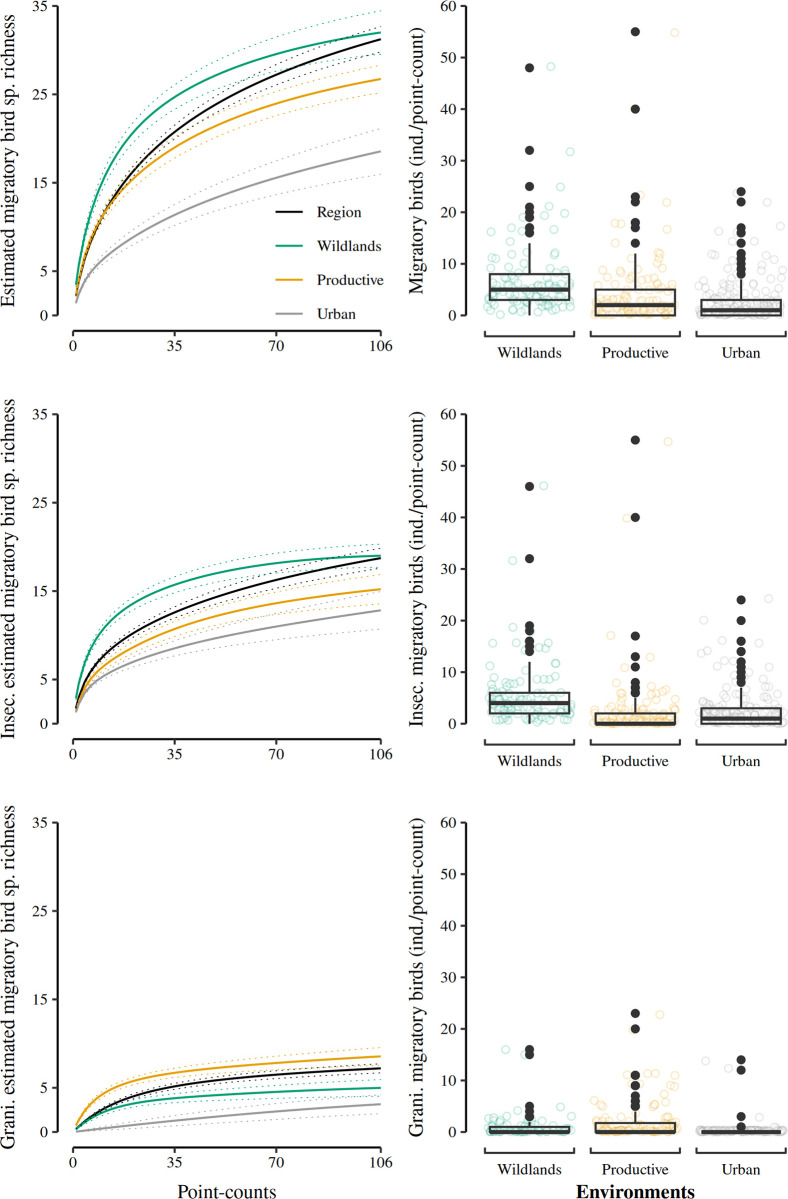
Migratory bird species richness and abundance in the three environments of El Bajío region. Left panels show incidence-based rarefaction estimated species richness (qD = 0) with their 84% confidence intervals for the region (black), and the three environments: wildlands (green), productive (yellow), and urban (gray). The right panels show boxplots comparing the abundance of migratory birds (ind./point-count) among the three environments. Jittered points represent the abundance recorded at each point-count. The top row of figures includes all migratory bird species, the central row presents only insectivorous bird data, and the bottom row shows data on granivorous migratory birds only.

The wildland environment had the highest estimated species richness of migratory birds (32; 29.8–34.2; ĉ: 0.99; 84% CI; see Fig 2). These values did not differ from the regional species richness (31.2; 29.8–32.7; ĉ: 0.96; 84% CI). Migratory bird species richness was lower in the productive environment (26.7; 24.5–29; ĉ: 0.97; 84% CI) than in the wildlands, with the urban environment presenting the lowest species richness of the three environments (18.6; 16–21.1; ĉ: 0.95; 84% CI). Migratory birds’ relative abundance differed among environments. The highest relative abundance occurred in the wildland environment, followed by the productive and the urban one (Table 1; Fig 2).

**Table 1:** Comparison of migratory bird abundances among the three environments we surveyed in El Bajio region: Wildlands, productive, and urban.

Response	Pairs	ratio (CI 95%)	SE	z ratio	p-value	
Mean individuals/point-count (40 m. radius-10 min.)	Wildlands / (Productive)	1.68 (1.15–2.45)	0.27	3.20	0.004	**
	Wildlands / Urban	2.5 (1.74–3.58)	0.38	5.94	p < 0.001	***
	Productive / Urban	1.49 (1.05–2.11)	0.22	2.69	0.020	*
Mean insectivorous ind./point-count (40 m. radius-10 min.)	Wildlands / Productive	2.46 (0.44–1.61)	0.44	5.00	p < 0.001	***
	Wildlands / Urban	2.25 (1.52–3.33)	0.38	4.83	p < 0.001	***
	Productive / Urban	0.91 (0.62–1.34)	0.15	-0.55	0.848	
Mean granivorous ind./point-count (40 m. radius-10 min.)	Wildlands / Productive	0.45 (0.19–1.02)	0.16	-2.28	0.059	
	Wildlands / Urban	4.31 (1.79–10.41)	1.62	3.89	p < 0.001	***
	Productive / Urban	9.68 (4.27–21.97)	3.39	6.50	p < 0.001	***

Data are presented for the whole migratory bird assemblage, and for insectivorous and granivorous birds separately. The values show a pairwise abundance ratio contrast done using a Posthoc Tukey test for each assessed set of relative abundances with a negative-binomial generalized linear model: all migratory birds, insectivorous and granivorous migratory birds. An extended summary of the models that show their estimated values is provided in Table A in S1 Text. [

*, p < 0.05

**, p < 0.01

***, p < 0.001].

**Fig 3:**
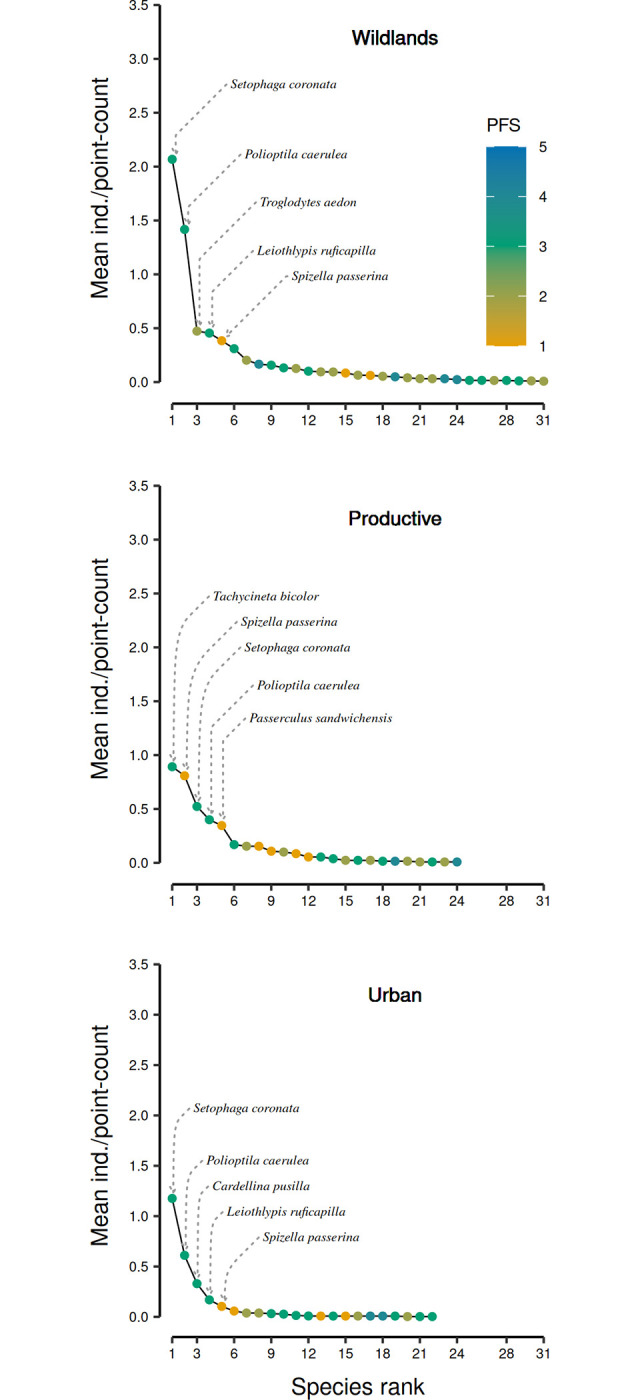
Rank-abundance plots of migratory bird assemblages surveyed in El Bajio region for wildlands (green), productive (yellow), and urban environments (gray). Assemblage structure varied among environments, with the wildlands migratory bird assemblage being more diverse and even than those present in the other two environments. Species rank values are presented in ascending order using each species’ mean relative abundance (ind./40 m radius point-count). Names of the five most dominant species are shown for each environment. Model result values are shown in Tables B and C in S1 Text.

Migratory bird assemblage structures differed among environments (Fig 3; Table B and C in S1 Text). *Setophaga coronata* and *Polioptila caerulea* were among the five most dominant species in the three environments of the urban region (Fig 3). Wildlands and urban environments shared most of their dominant species identities and ranks (4 out of five: *Setophaga coronata*, *Polioptila caerulea*, *Leiothlypis ruficapilla* and *Spizella passerina*). These four shared species represented 52.1% and 79.3% of the total bird individuals in the wildland and the urban environments respectively (Fig 3). Despite this peculiar similarity between these environments, wildlands presented a less dominated assemblage than urban areas, due to the different number of rare species (26 vs. 17 species, respectively). The productive environment differed in the composition and rank order of its most abundant species from the other two environments, with its five most dominant species standing for 73.6% of the abundance (Fig 3). Of those, the most dominant species was *Tachycineta bicolor*, followed by *Spizella passerina*, *Setophaga coronata*, *Polioptila caerulea*, and *Passerculus sandwichensis* (Fig 3).

**Fig 4:**
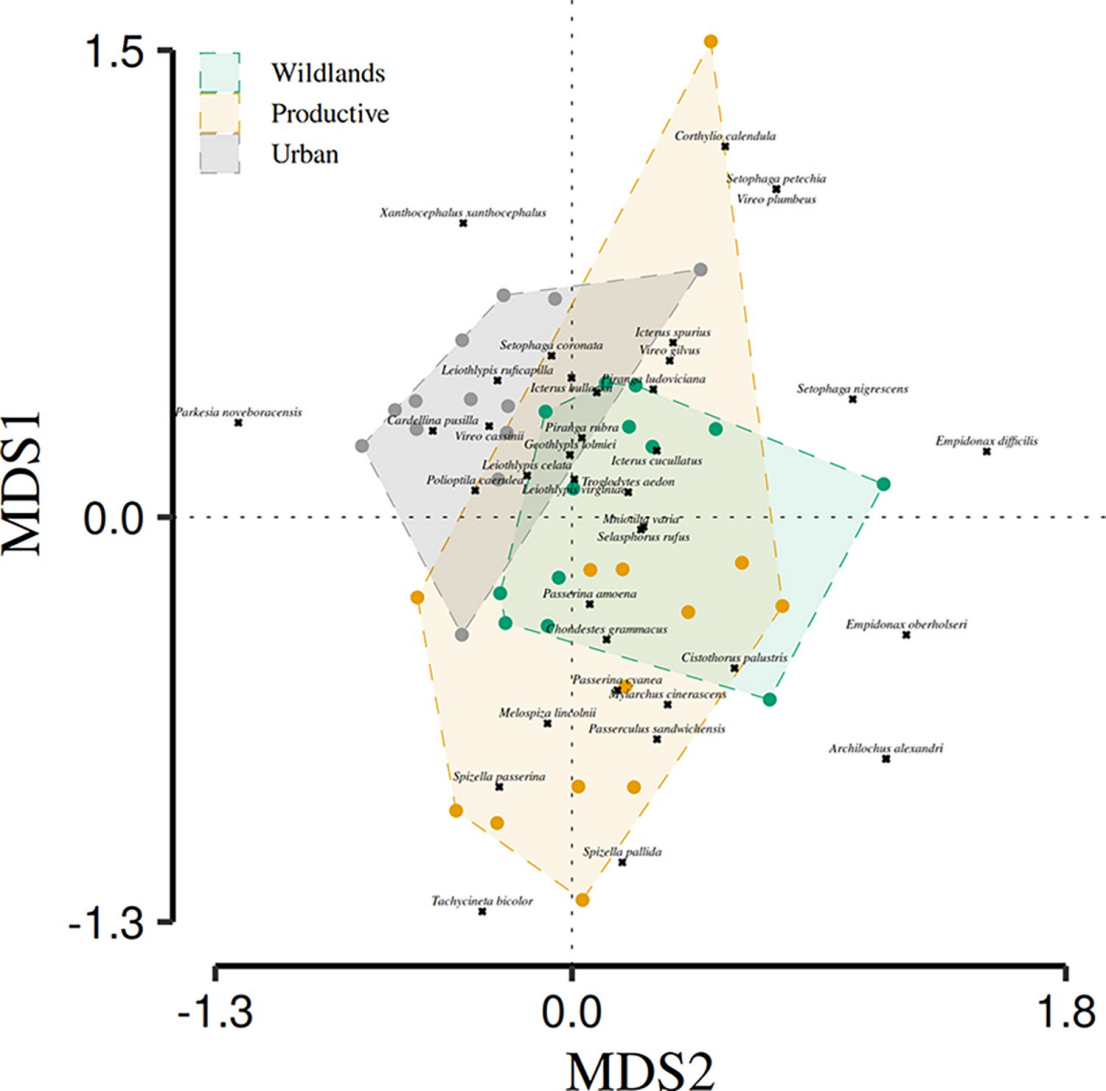
Multidimensional analysis of migratory bird assemblages composition in each environment surveyed in El Bajío region (wildlands: Green, productive: Yellow, and urban: Gray). Each point represents the assemblage found in each environment of the sampled thirteen localities. Composition of migratory bird assemblages was assessed using the Bray-Curtis beta diversity index processed by an MDS analysis. Black squares represent migratory bird species. An Adonis analysis showed that assemblage composition differed among the three environments.

Migratory bird assemblage composition also differed among the three environments (Table 2; Fig 4). The MDS and the adonis test showed the existence of three clusters. Each cluster was related to one environment, and while there was some overlap among the three clusters, all assemblage differed (Table 2; Fig 4). Wildlands exhibited unique species that did not occur in the other two environments, such as two hummingbirds (*Archilochus alexandri* and *Calothorax lucifer*). However, this environment also shared some species with the productive and the urban environments. These species were related to the granivore and insectivore trophic guilds, respectively.

**Table 2:** Comparison of the migratory bird assemblage composition among the three environments surveyed in El Bajio region: Wildlands, productive, and urban.

Pairs	SumsOfSqs	F value	p value	
Wildlands / Productive	1.146	5.066	0.003	**
Wildlands / Urban	1.125	6.414	0.003	**
Productive / Urban	1.005	5.262	0.006	**

We show the values of the adonis pairwise posthoc test, over the processed Bray-Curtis index by the MDS. The comparison was done by using each of the thirteen surveyed locations grouped by their respective environment. [

*, p < 0.05

**, p < 0.01

***, p < 0.001].

The phi coefficient analysis determined relevant associations of particular migratory bird species to the three sampled environments (Table 3). Six species were associated with wildlands (*Geothlypis tolmiei*, *Leiothlypis celata*, *Leiothlypis ruficapilla*, *Piranga ludoviciana*, *Polioptila caerulea*, and *Troglodytes aedon*). Three were associated with the productive environment (*Passerculus sandwichensis*, *Spizella pallida*, and *Spizella passerina*). One species was associated with both wildlands and productive environments (*Melospiza lincolnii*; Table 3). No species were associated with the urban environment.

**Table 3:** Phi coefficient (Φ) relevant associations of migratory bird species to our three surveyed environments in El Bajío region (wildlands, productive and urban).

Migratory bird species	Environmnets to which species is associated	Φ value	p value	
*Geothlypis tolmiei*	Wildlands	0.535	0.01	*
*Leiothlypis celata*	Wildlands	0.411	0.02	*
*Leiothlypis ruficapilla*	Wildlands	0.466	0.01	*
*Piranga ludoviciana*	Wildlands	0.503	0.02	*
*Polioptila caerulea*	Wildlands	0.715	0.01	*
*Troglodytes aedon*	Wildlands	0.717	0.01	*
*Melospiza lincolnii*	Wildlands & Productive	0.409	0.02	*
*Passerculus sandwichensis*	Productive	0.57	0.01	*
*Spizella pallida*	Productive	0.404	0.04	*
*Spizella passerina*	Productive	0.409	0.03	*

There were no relevant associations of species to urban environments. [

*, p < 0.05

**, p < 0.01

***, p < 0.001].

Insectivorous and granivorous were the most diverse migratory bird trophic guilds. The estimated species richness of the insectivorous tropic guild reached its highest value in wildlands (19; 17.5–20.5; ĉ: 1; 84% CI; Fig 2), being similar to the estimated species richness of this trophic guild in the regional pool (18.7; 17.6–19.9; ĉ: 0.97; 84% CI). The productive and urban environments had statistically similar migratory birds’ insectivorous species richness (15.2; 13.1–17.4; ĉ: 0.98 and 12.8, 10.8–14.9; ĉ: 0.96; 84% CI, respectively; Fig 2) and abundances (Fig 2; Table 1).

In the case of the granivorous trophic guild, its estimated species richness reached its highest value in the productive environment (8.6; 7.4–9.7; ĉ: 0.98; 84% CI), being similar to the estimated species richness value for the regional pool for this trophic guild (7.2; 6.6–7.8; ĉ: 0.95; 84% CI; Fig 2). The estimated granivorous species richness was similar for wildlands and urban environments (5; 3.9–6.1; ĉ: 0.97 and 3.1; 2.1–4.2; ĉ: 0.52; 84% CI; Fig 2). Migratory granivorous species abundance reached its highest values in both wildlands and productive environments and its lowest value in the urban one (Fig 2; Table 1).

**Fig 5:**
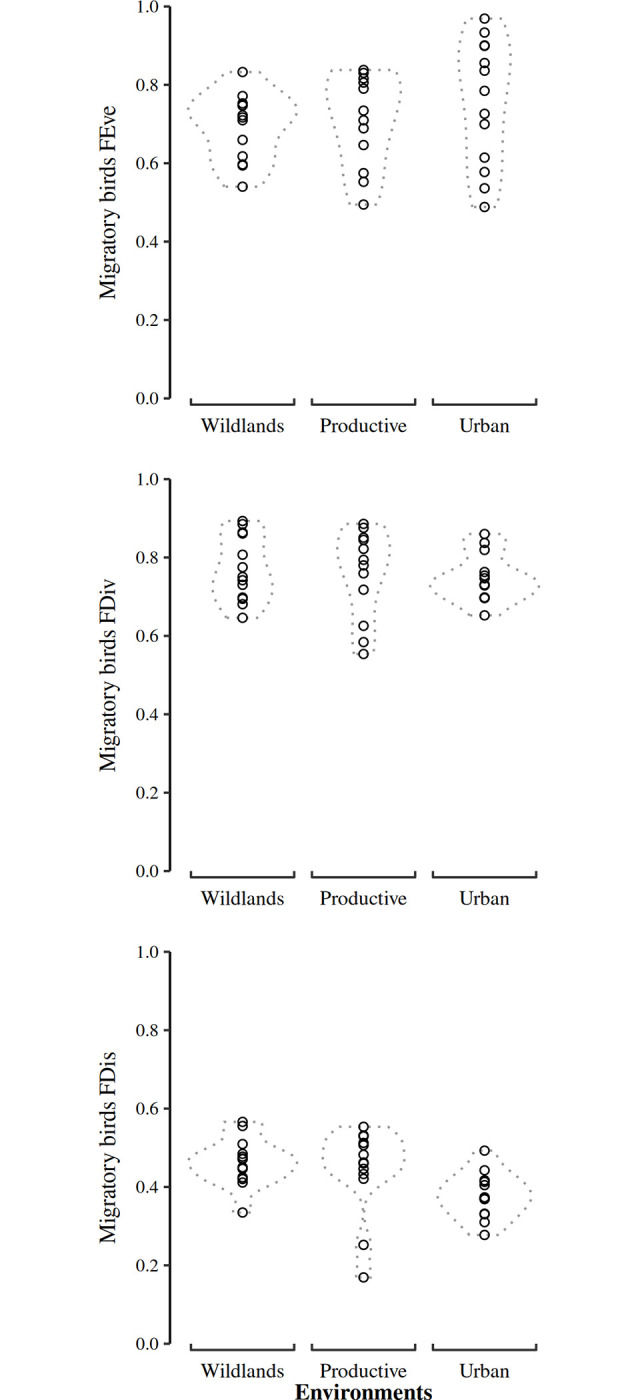
Migratory bird functional diversity indexes contrasted by our three surveyed environments in el Bajio region. The indexes FEve (top), FDiv (mid), and FDis (bottom) were calculated from categorical and continuous traits of each recorded species and processed by the Gower distance. We only found a difference in FDis values between wildlands and urban environments. The result values summaries of all Generalized Linear Models and their respective pairwise Posthoc Tukey tests are shown in Table D and E in S1 Text, respectively.

**Fig 6:**
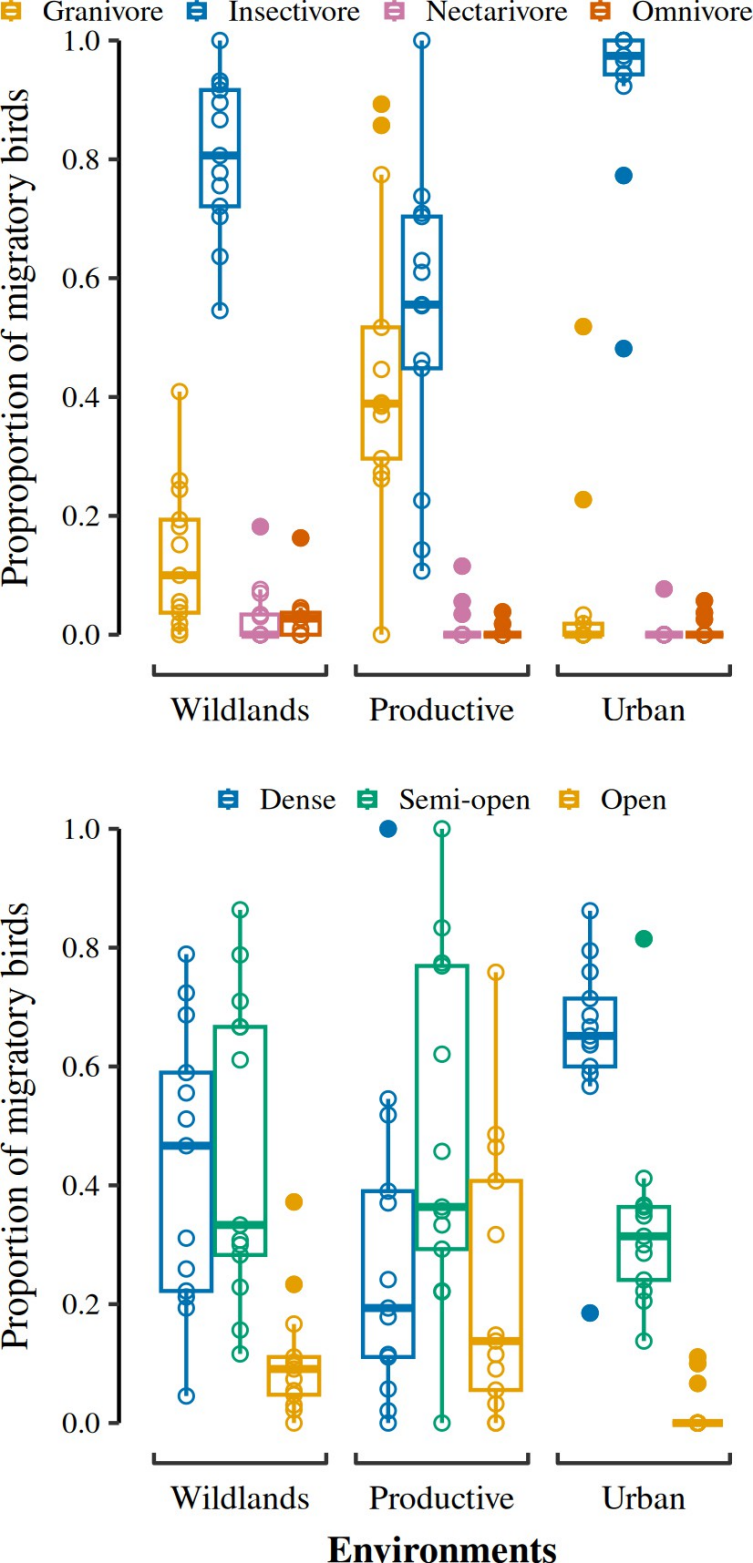
Proportions of individuals per trophic guild (top) and habitat preference categories (bottom) in the migratory bird assemblages of the three surveyed environments in El Bajío region. The insectivorous trophic guild presented a higher proportion of individuals both in wildlands and urban environments, while in the productive environments, granivorous and insectivorous birds had similar proportions of individuals. The proportion of individuals whose species were associated to dense habitats was higher in the urban environment, while species preferring open habitats showed a lower proportion of individuals both in this environment and wildlands. There were no differences among the proportion of individuals with preferences for different habitat densities in the productive environment. The result values summaries of all Generalized Linear Models that assess the categorical functional traits and their corresponding pairwise Posthoc Tukey test values are shown in Table F in S1 Text and S1 Table, respectively.

The Gower distance-based values on migratory bird functional traits did not show differences among environments in FDiv and FEve, but they did in FDis (Fig 5; Table D in S1 Text). The difference in FDis was due to higher values of trait dispersion in wildlands, relative to the urban environment (Table E in S1 Text). The proportion of insectivorous individuals in the assemblage was higher in wildlands and urban environments, with the other trophic guilds occupying similarly low proportions of the assemblage (Fig 6; see Table F in S1 Text and S1 Table). The productive environment had a similar proportion of insectivorous and granivorous bird individuals in its migratory bird assemblage (Fig 6; S1 Table). Gleaning was the dominant foraging behavior in all environments (see Table F in S1 Text and S1 Table).

The proportion of individuals by their preferred habitat density (dense, semi-open, and open) showed differences among environments (Fig 6; Table F in S1 Text). In wildlands, the proportions of individuals with dense and semi-open habitat preferences were equal and higher than those with open habitat preferences (S1 Table). In the productive environment, there were no differences in the proportion of migratory birds that preferred different habitat densities (Fig 6; S1 Table). However, this environment exhibited the highest values of the proportion of migratory birds that preferred semi-open and open habitats. In the urban environment, the preferred habitat densities had different proportions of individuals; the preference for dense habitat had the highest proportions of migratory birds, followed by the preference for semi-open, and open habitats (Fig 6; S1 Table). Moreover, the proportion of migratory birds with dense habitat preference was highest in urban areas relative to the wildlands and productive environments. Preference for semi-open and open habitat densities were similar among environments (Fig 6; S1 Table).

**Fig 7:**
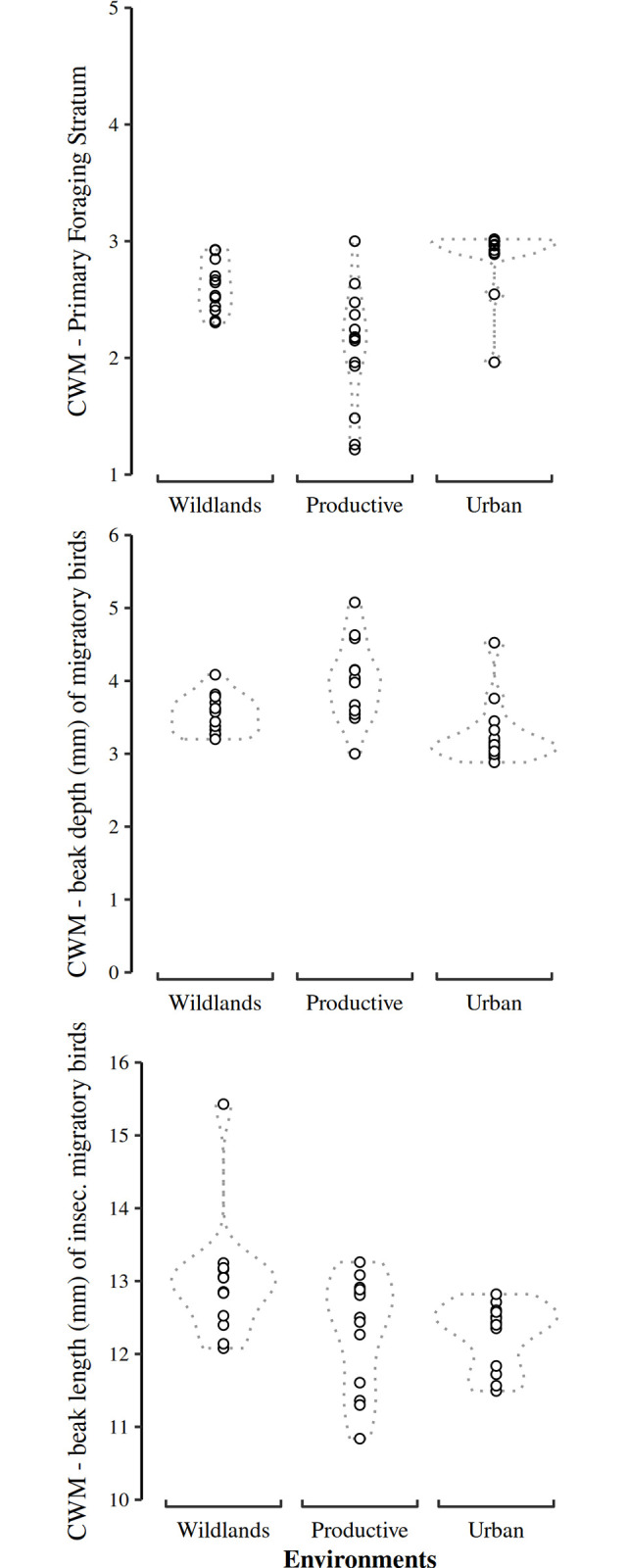
Comparison of the Community Weighted Mean (CWM) values of the Primary Foraging Stratum (PFS; top), beak depth (mm; center), and beak length (mm; bottom) of insectivorous migratory birds surveyed in the three environments of El Bajío. PFS values were higher in wildland and urban than in productive environment. Productive environment had the highest beak depths. The beak length of insectivorous migratory birds was lower in urban environments than in wildlands. The result values summaries of the Generalized Linear Models that assess the continuous functional traits and their corresponding pairwise Posthoc Tukey test values are shown in Table D and E in S1 Text, respectively.

The CWM values of the Primary Foraging Stratum (PFS) were higher in wildland and urban environments, relative to the productive one (Table E in S1 Text; Fig 7). Culmen length was smaller in the urban environment relative to wildlands (Table E in S1 Text; Fig 7). Beaks were wider in productive environments compared to the urban one. Beak depths were higher in productive environments (Table E in S1 Text; Fig 7). Among environments, migratory bird assemblages were similar in their body mass, tarsus length, hand wing index, and tail length (Table D in S1 Text). The contrasts among environments only considering insectivorous migratory birds showed that the pattern of higher CWM values of the PFS and shorter CWM beak lengths from culmen in urban environments relative to wildland environments persisted (Table E in S1 Text; Fig 7). Beak width values for insectivorous migratory birds were similar among environments (Table E in S1 Text).

## Discussion

Our study reveals that El Bajío supports a diverse assemblage of migratory birds during the winter months. This regional assemblage is dominated by a group of species that exhibit resilience to the anthropogenic transformation of their habitats and are present in the three environments. Notably, there are significant differences in the species richness, abundance, and composition of migratory bird assemblages among the three environments. Wildlands hosted migratory bird assemblages that presented higher species richness and functional diversity than urban environments. We propose that these differences in migratory bird assemblage characteristics result from a regional environmental filtering process. This filtering process is shaped by the functional traits of migratory bird species, the specific habitat characteristics of each environment, and resource constraints observed in both the productive and urban environments.

In this section, we first examine how migratory bird assemblages respond to the transformation of wildlands to productive areas, and urban environments by comparing their species richness, abundance, and composition. Second, we examine the differences in the structure of migratory bird assemblages among our studied environments, emphasizing the shared species. Third, we delve into the functional traits present in the assemblages of the three environments. Finally, we discuss the implications of our findings for conserving migratory birds in this critical overwintering region of the continent.

### Changes in migratory bird assemblages associated with land use change in El Bajío

As a result of 3,000 years of human management, our studied region presents one of the lowest levels of ecosystem integrity in the country [83]. Associated to the long history of intense human activities in this region, most of the migratory bird species we recorded do not present conservation problems [66]. Only one species, the long-distance migratory hummingbird *Selasphorus rufus* is considered as near threatened (IUCN red list). Interestingly, this species was only present on wildlands, the environment that presented the highest number of migratory species in the region.

The wildland patches of El Bajío contain a significant number of migratory bird species existing in other areas of Western Mexico with similar environmental characteristics (same altitudinal and latitudinal range; see S1 Fig; [84]). In this environment, we recorded a number of migratory bird species that did not differ from the calculated species richness for the regional pool. Most of the species that we only found in this environment prefer dense and semi-open habitats, and are associated with forests and shrublands as their primary habitat type. Additionally, this group of migratory birds exclusive to wildlands may suggest a shared low tolerance for human activity. However, migratory bird species unique to the wildlands of El Bajío, such as *Turdus migratorius* and *Geothlypis tolmiei* overwinter in neotropical cities of other regions of Mexico (S1 Fig and S1 Dataset; [42, 44, 84]), which suggest that the same species can respond differently to human activities depending on the regional context. These results indicate that despite its long historical alteration by humans, the wildlands of El Bajío are an essential habitat for overwintering non-endangered migratory birds today [32, 85, 86].

El Bajío shows two different trajectories of land use change that have affected migratory bird assemblages. The first one is the transformation of wildlands into productive areas. This transformation started millennia ago with the arrival of agriculture [87], and has undergone an important increment in recent centuries through the opening of extensive areas for intensive cultivation (Fig 1A and 1B; [49]). This first environmental change due to human activities generated essential changes in the assemblages of migratory birds, leading to a 40% species loss (13 species), with most of the missing species being those associated with semi-open and dense habitats [68]. Yet, productive environments offered new habitat conditions that allowed the arrival of eight migratory species with open habitat preferences to this region (21.8% of the total species richness observed in wild environments). These species could have invaded El Bajío from the grasslands of the Central Altiplano located north of it (≈100 km from our study area). The combination of species losses and turnover generated an assemblage that is 24% (Bray-Curtis: 0.22–0.26; CI 95%) similar to the one present before this agricultural land use change occurred.

The migratory bird assemblage of the productive environment also differed in its abundance in relation to wildlands, presenting 40% fewer individuals. This reduction in abundance was not associated with the loss of some species that accompanied the conversion of wildlands to productive environments. These species were already present in low abundances in wildlands, representing only 8.2% of the total migratory bird abundance in that environment. However, it was caused by a reduction in the number of individuals of two insectivorous species that were abundant in wildlands: *Setophaga coronata* and *Polioptila caerulea* that together represent 51.7 and 22.9% of the total abundance in wildlands and productive environments, respectively (Fig 3). The abundance difference of these species in these environments suggests that crucial resources may limit their densities in productive areas [88]. Interestingly, while some open-habitat species were dominant in the productive environment, their numbers did not compensate for the abundance loss compared to wildlands. While the productive environment of El Bajío offers opportunities for the arrival of open habitat migrant birds, it harbors assemblages that present lower species richness and abundance values, limiting their conservation potential value in the region [27].

The second trajectory of land use change in El Bajío is associated with urbanization transforming productive environments [49, 88]. This land use transition modifies the assemblage of migratory birds by losing 42.3% of the species that use the open areas (11 species). These species represent 39.5% of the migratory bird abundance in the productive environments of our study region, indicating that urban areas offer different habitat elements and food resources to this group of birds [47, 89]. For example, migratory birds in urban environments are strongly associated with green infrastructure like large street trees, or parks where trees are common habitat elements [36, 38, 42]. The reduction in bird abundance caused by the urbanization of productive environments was mainly associated with the loss of two species of migratory birds from open areas that were exclusive to, and abundant, in productive environments. These two species were *Tachycineta bicolor* and *Passerculus sandwichensis*, which together comprised 30.7% of the total abundance in productive environments. These results also suggest that the lower abundance of migratory birds in urban areas may be caused by reduced areas of high quality urban habitat and limited food resources in this environment [90].

Urbanization processes have also occurred in areas that used to be covered by native vegetation [89]. While this land use change occurs less frequently, in the last half century this process has generated housing suburbs of different socioeconomic levels around the largest cities of the region (e.g., Morelia, Irapuato, Moroleon-Uriangato, and Salamanca; [91]). This transformation of wildlands due to urbanization represents a loss of 43% of the species of the migratory bird assembly. Their populations also declined, as wildlands had 250% more individuals. These declines in species richness and abundance resembles those previously described by MacGregor-Fors [36] for the city of Morelia.

Interestingly, the urban migratory bird assemblage shared three of the 13 species present in wildlands that were absent in productive environments. This indicates that some migratory birds associated with wildlands are able to use urban environments [38]. Zuckerberg [92] observed that during their stage of long distance movements, migratory birds present a higher plasticity in habitat choice than during their breeding period, with some species favoring urban environments. Furthermore, during the winter, urban areas can be used by some migratory birds considered as interior forest specialists [38]. Our results, together with those from other studies, indicate that urban green features, like large trees, can offer migratory birds habitat elements that function similarly to some of those present in wildlands.

### Differences in assemblage structure among environments

The main differences in the migratory bird assemblages of the three environments were associated with: 1) changes in the abundances of common species shared among the three environments; and 2) the presence of some species that take advantage of particular habitat elements and resources offered by productive and urban environments. Similar mechanisms have been suggested to explain the spatial distribution of migratory birds in other Neotropical human-transformed regions [22, 24, 35]. For example, the abundance of specific food items, like grain or small arthropods, have been associated to higher densities of granivorous and insectivorous migratory birds respectively [27, 30, 32]. Additionally, birds from these two trophic groups benefit differentially from complex vegetation, with granivores depending on areas that present a dense understory with a high diversity of herbaceous plants, and that present low numbers of trees [27, 30, 92]. While insectivorous birds increase their species richness and abundances in response to the complexity of the canopy [22, 36, 42, 44].

While migratory bird assemblages differed among environments, they shared an important number of species. This shared group included 15 species, and represented 34% of the regional pool of migratory bird species at El Bajío. Shared species constituted 47% of the wildlands bird assemblage, while these values were larger in productive and urban environments (57% and 68% respectively). Most of the shared species were small insectivorous birds, mainly warblers (Fig 3 and S1 Dataset). These species have been stated to have a broad capacity to take advantage of a large diversity of habitats [26–28]. They have also been mentioned as being common or abundant in Neotropical urban bird assemblages [42].

The core group of migratory bird species shared among the three environments includes dominant species that represent 82% of the total regional abundance (Fig 3). While the shared species included the most abundant species, it is essential to notice that they differed in their abundance among environments, defining the structure of the different bird assemblages. Species abundance differences among environments have been associated with a change in the abundance of food resources and the quality of habitat patches in productive and urban environments [38]. It has also been associated with a gradient of human activity [93]. Assessing the role that habitat elements and human activity have on migratory bird behavior and habitat use offers important insights to manage this group of birds in human modified environments. In consequence, we recommend future studies to evaluate the role that trees (species involved, their architecture, and food resources), crops (type and managing activities), and human activity (human presence, noise, mobility) play on the behavior and ecology of migratory birds in both productive and urban overwintering environments [14].

### Functional traits of migratory birds in El Bajío

The regional migratory bird assemblage was primarily composed of insectivorous species, followed in importance by granivorous birds. This result is consistent with findings from other Neotropical overwintering regions [27, 28, 32]. Migratory bird assemblages varied in specific functional traits among the region’s studied environments [12, 93]. However, the core of species shared among environments (see above) suggests the existence of critical common functional traits. As a result in today’s landscape, any of the region’s environments can maintain the most abundant functional traits of the regional pool of migratory birds.

The core of shared species was characterized by small body-size insectivorous birds that engage in gleaning foraging behavior, and show preferences for dense and semi-open habitats. These species have been reported as typical winter inhabitants of Neotropical wildlands [27, 28, 32, 92], urban areas [38, 42], and productive environments [27]. Their presence and abundance in anthropogenic environments have been associated with a complex vegetation structure that includes a high density of trees, and an intricate lower stratum of shrubs and herbs [27, 38, 43]. While this core of species has the potential to maintain stable populations in anthropogenic environments [43, 85], it is crucial to understand their interactions with specific habitat elements, such as specific trees and shrub species, to perpetuate their populations in the future.

Other species in this core group included sparrows, cardinals, an icterid, and a hummingbird (see S1 Fig and S1 Dataset). This diverse collection of trophic groups of migratory birds in productive and urban environments highlights the broad spectrum of resources available to them in human-altered sites [94]. This high trophic diversity could be related to the presence of flowers and fruits in urban sites, and seeds in productive environments [27, 30, 31, 35, 92]. Additionally, human-modified habitats present novel habitat features, like buildings, cables, poles, and other structures, that help support bird assemblages associated with structurally complex habitats [39, 41, 95]. The presence of a surplus of water in human-altered environments during the cold-dry season of the year, when water is limiting, could also promote the presence of a diverse array of trophic guilds in these environments [17, 44, 95, 96].

The differences in migratory bird assemblages across environments, based on their functional traits, allow us to identify crucial environmental features that can act as opportunities or limitations for migratory birds. Wildlands presented abundant insectivorous birds, with our findings confirming the prevalence of this guild in Neotropical wildland assemblages [27, 28, 32]. However, the ten migratory bird species we only recorded on wildlands were diverse in their diets (nectarivores, insectivores, and omnivores) and presented semi-open and dense habitat preferences (S1 Fig and S1 Dataset). Their presence indicates an affinity for a complex vegetation structure that includes mature trees and several layers of vegetation (herbs and shrubs) while simultaneously offering semi-open spaces combined with dense understory areas [32, 38].

The migratory bird assemblage of productive environments was functionally characterized by a balanced proportion of the abundance of insectivorous and granivorous birds, and balanced abundances by habitat preferences (dense, semi-open, and open habitats). However, the seven migratory birds exclusively recorded in productive environments had open and semi-open habitat preferences. Their functional traits underscore the importance of open spaces for aerial foraging in productive environments (e.g., *Tachycineta bicolor*) while also promoting birds that forage for seeds on the ground (e.g., *Passerculus sandwichensis*; [27, 30, 31].

Our results indicate that the productive environment harbored the regional pool of migratory granivorous birds. Similarly, granivorous migratory birds have been described as abundant in intensively managed crops during their overwintering period in other regions of the continent [27, 30, 92]. Associated with this, the migratory bird assemblage of productive environments had the highest beak depth and the lower primary foraging stratum of our studied region. These functional traits indicate the importance of crop-related food resources and habitat characteristics of productive environments for some migratory bird species [27, 31, 35].

We also found that insectivorous birds had similar abundances between productive and urban environments. Both environments possess scattered tall trees that benefit this trophic group. However there are important differences among the way trees are distributed in space in these two environments [97]. In the productive environment, trees are primarily utilized as living fences marking property limits and being surrounded by crops. In urban environments trees are mostly concentrated in parks, and scattered mainly along streets. Our results indicate the importance of living fences and urban trees in Western Mexico’s, as they increase the vegetation complexity of intensely human modified environments, offering opportunities for birds that do not feed on seeds or use the ground for foraging [27, 30, 31, 97].

We were surprised to find a lack of migratory granivorous species in the urban assemblage. This is remarkable if we consider that resident urban bird assemblages are typically dominated by granivorous species, suggesting the presence of abundant food resources for this trophic guild [8, 98–100]. We propose three non-mutually exclusive hypothesis to explain the absence of granivorous migratory birds in the urban environment of El Bajío: 1) competitive exclusion by abundant non-migratory granivorous birds, which includes exotic species like the House sparrow (*Passer domesticus*), a species that is aggressive towards smaller granivorous bird species [8, 100]; 2) the lack of understory vegetation in urban environments that could limit grassland migratory granivorous species that forage and hide from predators among dense herbs [93, 97]; and 3) a lack of behavioral and physiological traits in migratory granivorous birds that prevent them from tolerating the urban stressors associated to human activity [39, 41, 101].

The lack of granivorous birds in the urban environment generates an assemblage dominated by insectivorous birds. Interestingly, the migratory insectivorous species using urban areas were characterized by presenting dense habitat preferences, higher Primary Foraging Stratum values, and smaller beak dimensions than those species using the other environments (S1 Text; Fig 6). As hypothesized by Greenberg’s breeding currency hypothesis [28], the availability of small arthropods in the tree canopy and plant foliage could be enhanced in environments that presented depauperized bird assemblages, like urban areas [32, 43, 85]. This could promote an abundance of insectivorous migratory birds with short culmens like the one we found. The three migratory birds exclusively recorded in urban environments had dense and semi-open habitat preferences and were associated to water related habitats (*Parkesia noveboracensis*, *Setophaga petechia* and *Vireo plumbeus*; S4 Dataset). This opportunity could arise by the urban year-round water surplus [15, 44, 95]. The functional characteristics of the urban migratory bird assemblage of El Bajío suggests that urban areas can act as simplified forests with a high water surplus [43]. Thus, our results indicate that offering water sources and increasing tree abundance and vegetation complexity is essential for enhancing migratory bird populations in urban environments.

### Future opportunities for migratory birds in a Mexican urban region

The high spatial dispersal ability of migratory birds makes them an ideal model for understanding how different factors mold their assemblages in the human-modified landscapes. Our results indicate species composition differences among environments, supporting the hypothesis that migratory species are filtered by their functional traits in anthropic environments [19, 20, 31, 93]. Additionally, our results suggest that while some migratory species can use the three studied environments, their lower abundances in the human-modified ones are related to a limitation on the abundance of key resources [22, 32, 102–104]. The shared core of migratory bird species indicates that some habitat features, primarily tree density and size, positively contribute to their presence and abundance in any environment. This result offers a promising opportunity to manage this biological group in a highly modified region.

Sadly, human modification of the wildlands of El Bajío by agricultural activities like grain or legume production is not the worst scenario migratory birds face in West Mexico today. In the last decade, traditional crops (corn, wheat, oats, sorghum, alfalfa, and beans; [56]) have been replaced by blue agave cultivars (*Agave tequilana*) used for the production of tequila [105]; personal observations). Agave production is not only being conducted on flat areas that present irrigation, but also on the hillsides where the last remnants of wildlands are located [105]. Agave cultivars have a low vegetation complexity, lacking the presence of trees, shrubs, and herbs. As a result, this habitat only supports nine migratory bird species (Ceja-Madrigal and Schondube unpublished data). This change from previous crop systems could generate additional negative impacts on the migratory bird populations of this region. As a result, we recommend conducting studies on the effects of the dramatic expansion of blue agave production in Mexico on migratory bird populations.

While conservation efforts for migratory birds in this region should focus on maintaining wildland patches [106], it is crucial to understand that both productive and urban environments offer opportunities to promote their conservation inside highly human modified regions [107]. The accelerated rate of land use change to agave cultivars experienced by this region suggests that urban areas can act as an essential habitat for migratory bird species as this productive system expands over the landscape [105]. To achieve this, we need to manage cities so they can offer good quality habitat for migratory birds. The fact that several migratory birds can use urban areas for overwintering while maintaining good physical condition indicates the need to integrate these birds into Neotropical urban planning [14, 15, 43, 107]. Our results indicate that increasing habitat quality for migratory birds in cities could become crucial for their conservation, considering the transformation suffered by wildlands and productive environments in the Anthropocene.

## Supporting information

S1 Text Comparative analysis of migratory bird assemblages across three environments surveyed in El Bajío region: Wildlands, productive, and urban. The file presents the results of multiple analyses comparing migratory bird assemblages in wildlands, productive, and urban environments within the El Bajío region. The comparisons cover migratory bird abundance, rank-abundance relationships, functional diversity, and functional traits. Table A provides abundance estimates for the entire assemblage, as well as insectivorous and granivorous birds separately, using negative binomial generalized linear models. Table B displays rank-abundance plots using a linear model with log-transformed relative abundance, and Table C details pairwise comparisons via posthoc Tukey tests. Table D contrasts functional diversity indices (FDiv, FEve, FDis) and community-weighted means (CWM) of continuous functional traits (e.g., beak dimensions, body mass, foraging stratum) using Generilized Linear Models and Table E presents their pairwise comparisons via posthoc Tukey tests. Table F shows constrasts of categorical functional traits (trophic guilds, foraging behavior, and habitat density preferences) using generalized linear models.
(DOCX)

S1 Table Posthoc Tukey pairwise comparisons of migratory bird assemblages by their proportional abundances in the groups defined by their categorical functional traits of trophic guild, foraging behavior, and habitat density, and among the three environments surveyed in El Bajio region: Wildlands, productive, and urban. (CSV)

S1 Fig Migratory bird species recorded in our study and through citizen science (2018–2022) in Western Mexico [84]. The Venn diagram show species recorded in our study or by citizen science within similar altitudes (1700–2200 masl) and latitudes as the study site. Species from our study are categorized by the environment in which they were observed: non-urban wildlands, non-urban productive, and urban environments. Species found in more than one environment are show in the environment are shown in the intersections. Species recorded by citizen science were not observed during our study but were documented either within the study area (El Bajío) or in nearby regions of Western Mexico.
(PDF)

S1 Dataset Incidence and abundance data of migratory bird species across point counts recorded in our study. The dataset includes two tabs. The first tab, labeled as “Incidence”, contains an incidence table showing the frequency of migratory bird species recorded at each point count, with species sorted from highest to lowest frequency of records. Each column represents a species record vector to be used with the iNEXT function: iNEXT(data, datatype = "incidence_freq", conf = 0.84, q = 0, endpoint = 106, knots = 60). The second tab provides an abundance matrix, presenting the mean number of individuals of each migratory bird species observed in 40-meter radius point counts, categorized by habitat and location.
(ODS)
