# Supplementary material for: Migratory birds benefit from urban environments in a highly anthropized Neotropical region
Source: PLoS One. 2025 Jan 24;20(1):e0311290. doi: 10.1371/journal.pone.0311290 (PMC11760022; doi:10.1371/journal.pone.0311290)
Supplement: S1 Text — The file presents the results of multiple analyses comparing migratory bird assemblages in wildlands, productive, and urban environments within the El Bajío region. The comparisons cover migratory bird abundance, rank-abundance relationships, functional diversity, and functional traits. Table A provides abundance estimates for the entire assemblage, as well as insectivorous and granivorous birds separately, using negative binomial generalized linear models. Table B displays rank-abundance plots using a linear model with log-transformed relative abundance, and Table C details pairwise comparisons via posthoc Tukey tests. Table D contrasts functional diversity indices (FDiv, FEve, FDis) and community-weighted means (CWM) of continuous functional traits (e.g., beak dimensions, body mass, foraging stratum) using Generilized Linear Models and Table E presents their pairwise comparisons via posthoc Tukey tests. Table F shows constrasts of categorical functional traits (trophic guilds, foraging behavior, and habitat density preferences) using generalized linear models. (DOCX) [file pone.0311290.s001.docx]

S1A Table. Comparison of migratory bird abundances among the three environments we surveyed in El Bajio region: wildlands, productive, and urban.

| Response | term | Estimate | Std. Error | z value | p value |
| --- | --- | --- | --- | --- | --- |
| Mean individuals/point-count (40 m. radius - 10 min.) | (Intercept) | 1.91 | 0.12 | 16.16 | 0 |
|  | Productive | -0.52 | 0.16 | -3.2 | 0.001 |
|  | Urban | -0.92 | 0.15 | -5.94 | p < 0.001 |
| Mean insectivorous ind./point-count (40 m. radius - 10 min.) | (Intercept) | 1.72 | 0.13 | 13.27 | p < 0.001 |
|  | Productive | -0.9 | 0.18 | -5 | p < 0.001 |
|  | Urban | -0.81 | 0.17 | -4.83 | p < 0.001 |
| Mean granivorous ind./point-count (40 m. radius - 10 min.) | (Intercept) | -0.27 | 0.27 | -1 | 0.318 |
|  | Productive | 0.81 | 0.35 | 2.28 | 0.023 |
|  | Urban | -1.46 | 0.38 | -3.89 | p < 0.001 |

Data are presented for the whole migratory bird assemblage, and for insectivorous and granivorous birds separately. The values show the estimated migratory bird abundance in each environment and their comparison among environments assessed in each negative binomial generalized linear model.

S1B Table. Comparison of rank-abundance plots of migratory bird assemblages among the three environments surveyed in El Bajio region: wildlands, productive, and urban.

| Response | term | Estimate | Std. Error | t value | p value |
| --- | --- | --- | --- | --- | --- |
| Rank | (Intercept) | -0.069 | 0.062 | -1.111 | 0.27 |
|  | rank | -0.066 | 0.003 | -19.533 | p < 0.001 |
|  | Productive | 0.006 | 0.095 | 0.066 | 0.947 |
|  | Urban | -0.302 | 0.097 | -3.106 | 0.003 |
|  | rank:Productive | -0.027 | 0.006 | -4.424 | p < 0.001 |
|  | rank:Urban | -0.042 | 0.007 | -6.379 | p < 0.001 |

The comparison among environments was done with the linear model, with the log-transformed relative abundance [*, p < 0.05; **, p < 0.01; ***, p < 0.001].

S1C Table. Pairwise comparison of rank-abundance plots of migratory bird assemblages among the three environments surveyed in El Bajio region: wildlands, productive, and urban.

| Contrast | Estimate (CI 95 %) | SE | df | t ratio | p value |  |
| --- | --- | --- | --- | --- | --- | --- |
| Wildlands - Productive | 0.358 (0.245 – 0.47) | 0.047 | 71 | 7.59 | < 0.001 | *** |
| Wildlands - Urban | 0.878 (0.76 – 0.996) | 0.049 | 71 | 17.78 | < 0.001 | *** |
| Productive - Urban | 0.52 (0.397 – 0.644) | 0.052 | 71 | 10.06 | < 0.001 | *** |

The pairwise comparison was done using a Posthoc pairwise Tukey test on the values obtained with the linear model, with a log-transformed relative abundance [*, p < 0.05; **, p < 0.01; ***, p < 0.001].

S1D Table. Contrast of migratory bird assemblages among the three environments surveyed in El Bajio region: wildlands, productive, and urban, by the functional diversity mutitrait Gower indexes (FDiv, FEve, and FDis) and the Community Weighted Means (CWM) of continuous functional traits.

| Response | Term | Estimate | Std. Error | t value | p value |  |
| --- | --- | --- | --- | --- | --- | --- |
| FDiv | (Intercept) | 0.771 | 0.024 | 31.613 | p < 0.001 | *** |
|  | Productive | -0.014 | 0.035 | -0.385 | 0.703 |  |
|  | Urban | -0.024 | 0.035 | -0.7 | 0.489 |  |
| FEve | (Intercept) | 0.693 | 0.035 | 19.937 | p < 0.001 | *** |
|  | Productive | 0.014 | 0.05 | 0.276 | 0.784 |  |
|  | Urban | 0.062 | 0.049 | 1.271 | 0.212 |  |
| FDis | (Intercept) | 0.771 | 0.024 | 31.613 | p < 0.001 | *** |
|  | Productive | -0.014 | 0.035 | -0.385 | 0.703 |  |
|  | Urban | -0.024 | 0.035 | -0.7 | 0.489 |  |
| CWM - PFS | (Intercept) | 2.627 | 0.103 | 25.609 | p < 0.001 | *** |
|  | Productive | -0.545 | 0.145 | -3.757 | 0.001 | ** |
|  | Urban | 0.224 | 0.145 | 1.546 | 0.131 |  |
| CWM - body mass (g) | (Intercept) | 11.579 | 0.935 | 12.38 | p < 0.001 | *** |
|  | Productive | 1.346 | 1.323 | 1.018 | 0.316 |  |
|  | Urban | -1.237 | 1.323 | -0.935 | 0.356 |  |
| CWM - Beak length culmen (mm) | (Intercept) | 13.469 | 0.231 | 58.185 | p < 0.001 | *** |
|  | Productive | -0.782 | 0.327 | -2.388 | 0.022 | * |
|  | Urban | -0.834 | 0.327 | -2.549 | 0.015 | * |
| CWM - Beak width (mm) | (Intercept) | 3.432 | 0.07 | 48.812 | p < 0.001 | *** |
|  | Productive | 0.168 | 0.099 | 1.694 | 0.099 |  |
|  | Urban | -0.206 | 0.099 | -2.069 | 0.046 | * |
| CWM - Beak depth (mm) | (Intercept) | 3.544 | 0.121 | 29.328 | p < 0.001 | *** |
|  | Productive | 0.448 | 0.171 | 2.622 | 0.013 | * |
|  | Urban | -0.266 | 0.171 | -1.556 | 0.128 |  |
| CWM - Tarsus lenght (mm) | (Intercept) | 17.574 | 0.322 | 54.621 | p < 0.001 | *** |
|  | Productive | -0.333 | 0.455 | -0.732 | 0.469 |  |
|  | Urban | 0.224 | 0.455 | 0.492 | 0.625 |  |
| CWM - Hand wing index | (Intercept) | 23.223 | 1.109 | 20.949 | p < 0.001 | *** |
|  | Productive | 2.506 | 1.568 | 1.598 | 0.119 |  |
|  | Urban | -0.389 | 1.568 | -0.248 | 0.806 |  |
| CWM - Tail lenght (mm) | (Intercept) | 52.134 | 0.824 | 63.304 | p < 0.001 |  |
|  | Productive | 0.917 | 1.165 | 0.787 | 0.436 |  |
|  | Urban | 0.31 | 1.165 | 0.266 | 0.792 |  |
| CWM - Insectivorous PFS | (Intercept) | 2.866 | 0.031 | 92.044 | p < 0.001 | *** |
|  | Productive | 0.043 | 0.044 | 0.988 | 0.33 |  |
|  | Urban | 0.112 | 0.044 | 2.542 | 0.015 | * |
| CWM - Insectivorous beak lenght culmen | (Intercept) | 12.985 | 0.197 | 66.065 | p < 0.001 | *** |
|  | Productive | -0.669 | 0.278 | -2.408 | 0.021 | * |
|  | Urban | -0.715 | 0.278 | -2.574 | 0.014 | * |
| CWM - Insectivorous beak width culmen | (Intercept) | 3.295 | 0.061 | 53.818 | p < 0.001 | *** |
|  | Productive | -0.117 | 0.087 | -1.357 | 0.183 |  |
|  | Urban | -0.186 | 0.087 | -2.148 | 0.039 | * |

The contrasts among environments were done using Generalized Linear Models. The Gower distance-related indexes are functional divergence (FDiv), functional evenness (FEve) and functional dispersion (FDis). The Community Weighted Means (CWM) of continuous functional traits are the Primary Foraging Stratum (PFS), body mass (g), beak length culmen (mm), beak width (mm), beak depth (mm), tarsus length (mm), hand wing index, and tail length (mm). We also contrasted the Primary Foraging Stratum, the beak length and beak width from culmen, only considering insectivorous migratory birds.

S1E Table. Posthoc Tukey pairwise comparisons of migratory bird assemblages among the three studied environments in El Bajío region: wildlands, productive, and urban, over the Generalized Linear Models contrasting the multitrait gower index (FDiv, FEve, FDis) and the Community Weighted Means (CWM) of Continuous Functional Traits.

| Response | Contrast | Estimate (CI 95 %) | SE | df | t ratio | p value |  |
| --- | --- | --- | --- | --- | --- | --- | --- |
| FDiv | Wildlands-Productive | 0.014 (-0.073 – 0.1) | 0.035 | 35 | 0.385 | 0.922 |  |
|  | Wildlands-Urban | 0.024 (-0.06 – 0.109) |  |  | 0.7 | 0.765 |  |
|  | Productive-Urban | 0.011 (-0.076 – 0.097) |  |  | 0.301 | 0.951 |  |
| FEve | Wildlands-Productive | -0.014 (-0.137 – 0.109) | 0.05 | 35 | -0.276 | 0.959 |  |
|  | Wildlands-Urban | -0.062 (-0.183 – 0.058) |  |  | -1.271 | 0.421 |  |
|  | Productive-Urban | -0.049 (-0.171 – 0.074) |  |  | -0.97 | 0.601 |  |
| FDis | Wildlands-Productive | 0.02 (-0.057 – 0.098) | 0.032 | 36 | 0.641 | 0.799 |  |
|  | Wildlands-Urban | 0.086 (0.009 – 0.164) |  |  | 2.716 | 0.027 | * |
|  | Productive-Urban | 0.066 (-0.012 – 0.143) |  |  | 2.075 | 0.109 |  |
| CWM - PFS | Wildlands-Productive | 0.545 (0.191 – 0.9) | 0.145 | 36 | 3.757 | 0.002 | ** |
|  | Wildlands-Urban | -0.224 (-0.579 – 0.13) |  |  | -1.546 | 0.282 |  |
|  | Productive-Urban | -0.769 (-1.124 – -0.415) |  |  | -5.303 | p < 0.001 | *** |
| CWM-body mass (g) | Wildlands-Productive | -1.346 (-4.579 – 1.887) | 1.323 | 36 | -1.018 | 0.571 |  |
|  | Wildlands-Urban | 1.237 (-1.996 – 4.469) |  |  | 0.935 | 0.622 |  |
|  | Productive-Urban | 2.583 (-0.65 – 5.815) |  |  | 1.953 | 0.139 |  |
| CWM-Beak length culmen (mm) | Wildlands-Productive | 0.782 (-0.018 – 1.582) | 0.327 | 36 | 2.388 | 0.057 |  |
|  | Wildlands-Urban | 0.834 (0.034 – 1.635) |  |  | 2.549 | 0.039 | * |
|  | Productive-Urban | 0.053 (-0.748 – 0.853) |  |  | 0.161 | 0.986 |  |
| CWM-Beak width (mm) | Wildlands-Productive | -0.168 (-0.412 – 0.075) | 0.099 | 36 | -1.694 | 0.221 |  |
|  | Wildlands-Urban | 0.206 (-0.037 – 0.449) |  |  | 2.069 | 0.111 |  |
|  | Productive-Urban | 0.374 (0.131 – 0.617) |  |  | 3.763 | 0.002 | ** |
| CWM-Beak depth (mm) | Wildlands-Productive | -0.448 (-0.866 – -0.03) | 0.171 | 36 | -2.622 | 0.033 | * |
|  | Wildlands-Urban | 0.266 (-0.152 – 0.684) |  |  | 1.556 | 0.277 |  |
|  | Productive-Urban | 0.714 (0.296 – 1.132) |  |  | 4.178 | 0.001 | ** |
| CWM-Tarsus lenght (mm) | Wildlands-Productive | 0.333 (-0.779 – 1.445) | 0.455 | 36 | 0.732 | 0.746 |  |
|  | Wildlands-Urban | -0.224 (-1.336 – 0.888) |  |  | -0.492 | 0.875 |  |
|  | Productive-Urban | -0.557 (-1.669 – 0.555) |  |  | -1.224 | 0.447 |  |
| CWM-Hand wing index | Wildlands-Productive | -2.506 (-6.337 – 1.326) | 1.568 | 36 | -1.598 | 0.259 |  |
|  | Wildlands-Urban | 0.389 (-3.443 – 4.22) |  |  | 0.248 | 0.967 |  |
|  | Productive-Urban | 2.894 (-0.938 – 6.726) |  |  | 1.846 | 0.169 |  |
| CWM-Tail lenght (mm) | Wildlands-Productive | -0.917 (-3.764 – 1.93) | 1.165 | 36 | -0.787 | 0.713 |  |
|  | Wildlands-Urban | -0.31 (-3.157 – 2.537) |  |  | -0.266 | 0.962 |  |
|  | Productive-Urban | 0.607 (-2.239 – 3.454) |  |  | 0.521 | 0.861 |  |
| CWM-Insectivorous PFS | Wildlands-Productive | -0.043 (-0.151 – 0.064) | 0.044 | 36 | -0.988 | 0.589 |  |
|  | Wildlands-Urban | -0.112 (-0.22 – -0.004) |  |  | -2.542 | 0.04 | * |
|  | Productive-Urban | -0.068 (-0.176 – 0.039) |  |  | -1.554 | 0.279 |  |
| CWM-Insectivorous beak lenght culmen | Wildlands-Productive | 0.669 (-0.01 – 1.349) | 0.278 | 36 | 2.408 | 0.054 |  |
|  | Wildlands-Urban | 0.715 (0.036 – 1.395) |  |  | 2.574 | 0.037 | * |
|  | Productive-Urban | 0.046 (-0.633 – 0.725) |  |  | 0.166 | 0.985 |  |
| CWM-Insectivorous beak width culmen | Wildlands-Productive | 0.117 (-0.094 – 0.329) | 0.087 | 36 | 1.357 | 0.374 |  |
|  | Wildlands-Urban | 0.186 (-0.026 – 0.398) |  |  | 2.148 | 0.094 |  |
|  | Productive-Urban | 0.068 (-0.143 – 0.28) |  |  | 0.791 | 0.711 |  |

The posthoc Tukey tests were performed over their respective linear models. The Gower distance-related indexes are functional divergence (FDiv), functional evenness (FEve) and functional dispersion (FDis). The Community Weighted Means (CWM) of continuous functional traits are the Primary Foraging Stratum (PFS), body mass (g), beak length culmen (mm), beak width (mm), beak depth (mm), tarsus length (mm), hand wing index, and tail length (mm). We also contrasted the Primary Foraging Stratum, the beak length and beak width from culmen only considering insectivorous migratory birds. [*, p < 0.05; **, p < 0.01; ***, p < 0.001].

S1F Table. Contrast of migratory bird assemblages by their proportional abundances in the groups defined by their categorical functional traits of trophic guild, foraging behavior, and habitat density, and among the three environments surveyed in El Bajio region: wildlands, productive, and urban.

| Response | Term | Estimate | std.error | t value | p value |  |
| --- | --- | --- | --- | --- | --- | --- |
| Trophic guild (proportion) | (Intercept) | 0.131 | 0.037 | 3.544 | 0.001 | ** |
|  | Insectivorous | 0.675 | 0.052 | 12.905 | p < 0.001 |  |
|  | Nectarivorous | -0.098 | 0.052 | -1.881 | 0.062 |  |
|  | Omnivorous | -0.101 | 0.052 | -1.937 | 0.055 |  |
|  | Productive | 0.319 | 0.052 | 6.099 | p < 0.001 | *** |
|  | Urban | -0.07 | 0.052 | -1.333 | 0.185 |  |
|  | Insectivorous:Productive | -0.596 | 0.074 | -8.052 | p < 0.001 | *** |
|  | Nectarivorous:Productive | -0.336 | 0.074 | -4.541 | p < 0.001 | *** |
|  | Omnivorous:Productive | -0.345 | 0.074 | -4.656 | p < 0.001 | *** |
|  | Insectivorous:Urban | 0.187 | 0.074 | 2.526 | 0.013 | ** |
|  | Nectarivorous:Urban | 0.043 | 0.074 | 0.581 | 0.562 |  |
|  | Omnivorous:Urban | 0.049 | 0.074 | 0.665 | 0.507 |  |
| Foragin behaviour (proportion) | (Intercept) | 0.028 | 0.029 | 0.96 | 0.339 |  |
|  | Gleaner | 0.912 | 0.041 | 22.437 | p < 0.001 | *** |
|  | Hovering | 0.005 | 0.041 | 0.126 | 0.9 |  |
|  | Productive | 0.051 | 0.041 | 1.26 | 0.21 |  |
|  | Urban | -0.028 | 0.041 | -0.679 | 0.499 |  |
|  | Gleaner:Productive | -0.086 | 0.057 | -1.488 | 0.14 |  |
|  | Hovering:Productive | -0.068 | 0.057 | -1.186 | 0.238 |  |
|  | Gleaner:Urban | 0.082 | 0.057 | 1.426 | 0.157 |  |
|  | Hovering:Urban | 0.001 | 0.057 | 0.014 | 0.989 |  |
| Habitat density (proportion) | (Intercept) | 0.428 | 0.059 | 7.266 | p < 0.001 | *** |
|  | Density Open | -0.32 | 0.083 | -3.844 | p < 0.001 | *** |
|  | Density Semi open | 0.036 | 0.083 | 0.428 | 0.67 | * |
|  | Productive | -0.14 | 0.083 | -1.684 | 0.095 |  |
|  | Urban | 0.214 | 0.083 | 2.572 | 0.011 | * |
|  | Density Open:Productive | 0.264 | 0.118 | 2.242 | 0.027 | * |
|  | Density Semi open:Productive | 0.157 | 0.118 | 1.33 | 0.186 |  |
|  | Density Open:Urban | -0.301 | 0.118 | -2.552 | 0.012 | * |
|  | Density Semi open:Urban | -0.342 | 0.118 | -2.903 | 0.004 | ** |

The contrasts among categories of each functional trait and environments were done using Generalized Linear Models. We show the estimated values of their linear models. Their pairwise posthoc Tukey test are shown at S2 Table. The trophic guild categories included granivore, insectivore, nectarivore, and omnivore birds; the foragin behaviour categories included gleanin air-hawking, gleaning and hovering; habitat preference categories included Dense, semi-open and open. [*, p < 0.05; **, p < 0.01; ***, p < 0.001].
