# Supplementary material for: Migratory birds benefit from urban environments in a highly anthropized Neotropical region
Source: PLoS One. 2025 Jan 24;20(1):e0311290. doi: 10.1371/journal.pone.0311290 (PMC11760022; doi:10.1371/journal.pone.0311290)
Supplement: S1 Fig — The Venn diagram show species recorded in our study or by citizen science within similar altitudes (1700–2200 masl) and latitudes as the study site. Species from our study are categorized by the environment in which they were observed: non-urban wildlands, non-urban productive, and urban environments. Species found in more than one environment are show in the environment are shown in the intersections. Species recorded by citizen science were not observed during our study but were documented either within the study area (El Bajío) or in nearby regions of Western Mexico. (PDF) [file pone.0311290.s003.pdf]

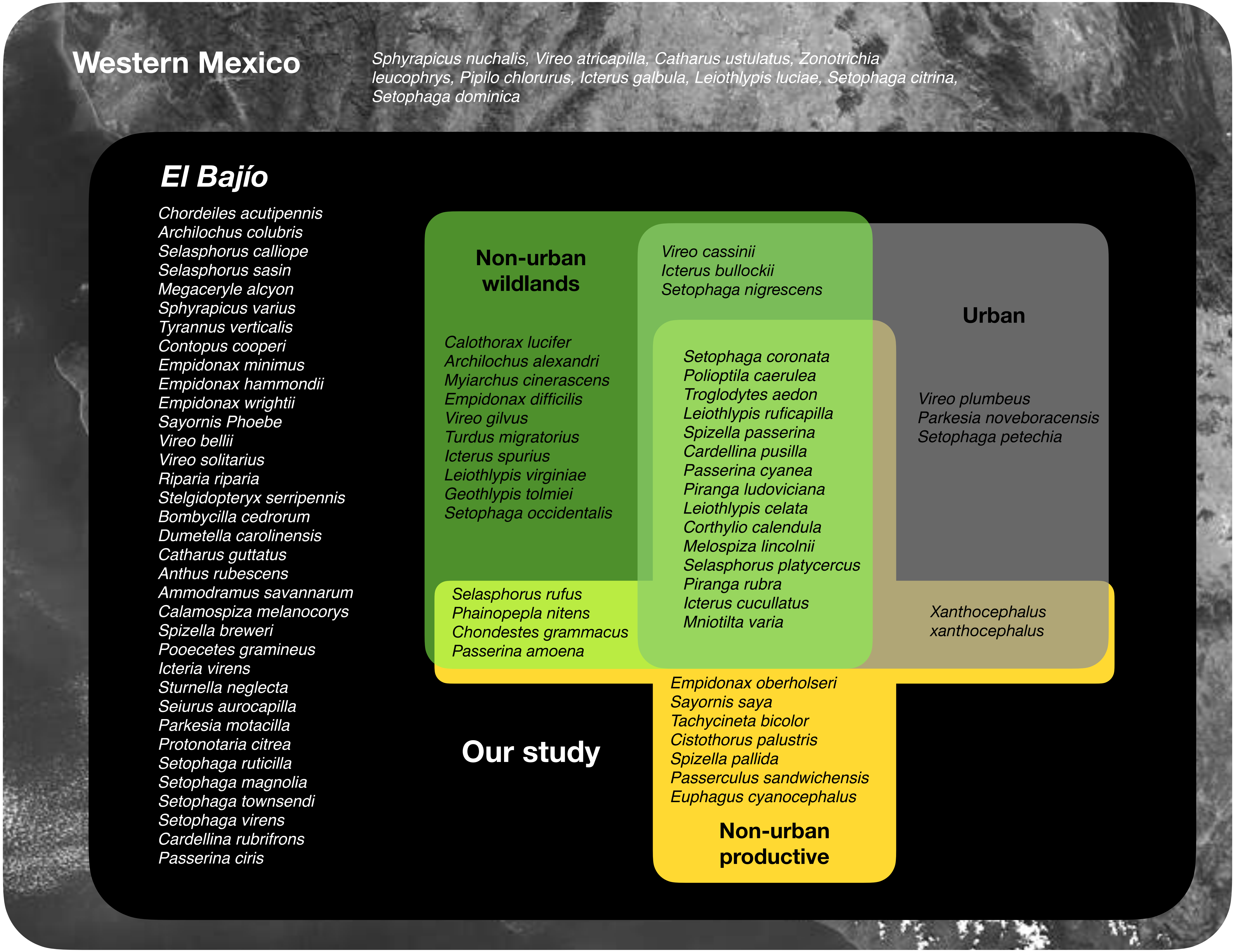
Western Mexico
Sphyrapicus nuchalis, Vireo atricapilla, Catharus ustulatus, Zonotrichia leucophrys, Pipilo chlorurus, Icterus galbula, Leiothlypis luciae, Setophaga citrina, Setophaga dominica
El Bajío
Chordeiles acutipennis
Archilochus colubris
Selasphorus calliope
Selasphorus sasin
Megaceryle alcyon
Sphyrapicus varius
Tyrannus verticalis
Contopus cooperi
Empidonax minimus
Empidonax hammondii
Empidonax wrightii
Sayornis Phoebe
Vireo bellii
Vireo solitarius
Riparia riparia
Stelgidopteryx serripennis
Bombycilla cedrorum
Dumetella carolinensis
Catharus guttatus
Anthus rubescens
Ammodramus savannarum
Calamospiza melanocorys
Spizella breweri
Pooecetes gramineus
Icteria virens
Sturnella neglecta
Seiurus aurocapilla
Parkesia motacilla
Protonotaria citrea
Setophaga ruticilla
Setophaga magnolia
Setophaga townsendi
Setophaga virens
Cardellina rubrifrons
Passerina ciris
Non-urban wildlands
Calothorax lucifer
Archilochus alexandri
Myiarchus cinerascens
Empidonax difficilis
Vireo gilvus
Turdus migratorius
Icterus spurius
Leiothlypis virginiae
Geothlypis tolmiei
Setophaga occidentalis
Vireo cassinii
Icterus bullockii
Setophaga nigrescens
Urban
Vireo plumbeus
Parkesia noveboracensis
Setophaga petechia
Setophaga coronata
Polioptila caerulea
Troglodytes aedon
Leiothlypis ruficapilla
Spizella passerina
Cardellina pusilla
Passerina cyanea
Piranga ludoviciana
Leiothlypis celata
Corthylio calendula
Melospiza lincolnii
Selasphorus platycercus
Piranga rubra
Icterus cucullatus
Mniotilta varia
Selasphorus rufus
Phainopepla nitens
Chondestes grammacus
Passerina amoena
Xanthocephalus xanthocephalus
Empidonax oberholseri
Sayornis saya
Tachycineta bicolor
Cistothorus palustris
Spizella pallida
Passerculus sandwichensis
Euphagus cyanocephalus
Non-urban productive
Our study
